# Preoperative radiotherapy for patients with rectal cancer: a risk factor for non-reversal of ileostomy caused by stenosis or stiffness proximal to colorectal anastomosis

**DOI:** 10.18632/oncotarget.20602

**Published:** 2017-09-01

**Authors:** Hongbo Zhu, Bingjun Bai, Lina Shan, Xiaowei Wang, Min Chen, Weifang Mao, Xuefeng Huang

**Affiliations:** ^1^ Department of Colorectal Surgery, Sir Run Run Shaw Hospital, School of Medicine, Zhejiang University, Hangzhou, China; ^2^ Key Laboratory of Biotherapy of Zhejiang province, Hangzhou, China

**Keywords:** rectal neoplasms, radiotherapy, anastomotic stenosis, permanent stoma, ileostomy

## Abstract

The influence of radiotherapy on permanent stoma and the bowel proximal to anastomosis was not well investigated. The current study aimed to analyze the effect of preoperative radiotherapy on colorectal anastomosis and incidence of non-reversal ileostomy. A total of 184 eligible patients with rectal cancer undergoing loop ileostomy were included. Patients were well selected by excluding some confounding factors and divided into two groups according to whether they received preoperative radiotherapy.

Patients with preoperative radiotherapy had higher incidence of non-reversal stoma (12.8%, *P =* 0.004) and stenosis or stiffness around anastomosis (21.1%, *P* < 0.01) including 13 patients with stenosis or stiffness proximal to anastomosis. Stenosis proximal to anastomosis was different from anastomotic stricture caused by surgery and could be described by imaging findings. Preoperative radiotherapy prolonged the interval to closure (*P =* 0.008) and was defined as a significant risk factor for permanent stoma (HR, 0.627; 95% CI, 0.405–0.973; *P =* 0.04) by multivariate Cox regression analysis. In conclusion, Preoperative radiotherapy increased incidence of non-reversal ileostomy and stenosis or stiffness proximal to anastomosis in rectal cancer patients with radical resection and diverting ileostomy.

## INTRODUCTION

Nowadays, multidisciplinary management has been applied in rectal cancer (RC) with the precise pre-operative staging, advancement of surgical technique, neoadjuvant chemoradiation therapy (NCRT) and adjuvant therapy, which results in an improved disease-free survival and overall survival [1, 2]. Patients with local advanced low or middle rectal cancer tend to choose NCRT and total mesorectal excision (TME) with sphincter preservation rather than abdominoperineal resection. NCRT offers plenty of advantages, including decreased tumor size, downgraded tumor stage even pathologic complete response (pCR), reduced local recurrence rate and more sphincter preservation [3].

Radical resection and diverting ileostomy is a standard surgical method for patients with rectal cancer after NCRT. A loop ileostomy is widespread used because of its convenient construction and closure to avoid some perioperative anastomotic complications [4, 5]. The complications including anastomotic leakage, fecal incontinence and fistula may result in an unreversed loop stoma or a permanent end colostomy, which greatly reduces the quality of patients’ life [6]. The risk factors associated with permanent stomas have been assessed previously [7–9]. Besides the perioperative complication, age, anastomotic level, local recurrence and radiotherapy are related to permanent stomas. Preoperative radiotherapy is likely to cause anastomotic leakage, anastomotic stenosis and stiffness around anastomosis, which would lead to permanent stomas [10]. However, the role of preoperative radiotherapy as a risk factor for permanent stomas still remains controversial [9, 11].

We performed a retrospective study with patients in appropriate control to identify the effect of preoperative radiotherapy on anastomosis, postoperative complications and permanent stomas rates.

## RESULTS

### Baseline characteristics

The baseline characteristics of all the patients are shown in Table 1. 184 patients were divided into two groups according to whether receiving preoperative radiotherapy (PRT+) or not (PRT-), which contained 133 and 51 patients, respectively. Patients without radiotherapy were significantly older than the other group (median age 62 and 60, respectively. *P* = 0.02). Higher ASA score was seen in the group of PRT− (*P* = 0.02). The primary tumors receiving radiotherapy had their ypT stages earlier than the T stages of tumor undergoing resection only (*P* = 0.003). Except the variables above, no differences were found between these two groups concerning sex, BMI, AJCC tumor stage, tumor location, level of anastomosis and underlying diseases.

**Table 1:** Baseline characteristics of patients from two groups

	All patients (*n =* 184)	PRT− (*n =* 51)	PRT+ (*n =* 133)	*P* value
Age	59.8	63.5 (38–83)	58.3 (26–78)	**0.02**^**a**^
Male gender	65.8%	70.6% (36)	63.9% (85)	0.39^b^
BMI	23.4	22.9 (17.3–33.6)	23.5 (17.5–35.8)	0.20^c^
ASA score				**0.02**^**b**^
I	42	15.7% (8)	25.6% (34)	
II	126	66.7% (34)	69.2% (92)	
III	16	17.6% (9)	5.3% (7)	
Tumor stage (AJCC)				0.65^b^
0/I	82	39.2% (20)	46.7% (62)	
II	61	35.3% (18)	32.3% (43)	
III	41	25.5% (13)	21.1% (28)	
T stage				**0.003**^**b**^
p/ypT1 or ypT0	49	19.6% (10)	29.3% (39)	
p/ypT2	41	27.5% (14)	20.3 (27)	
p/ypT3	75	31.4% (16)	44.4% (59)	
p/ypT4	19	21.6% (11)	6.0% (8)	
Tumor location				0.74^b^
≤ 5	39	19.6% (10)	21.8% (29)	
> 5	145	80.4% (41)	78.2% (104)	
Level of anastomosis				0.74^b^
≤ 4	126	66.7% (34)	69.2% (92)	
> 4	58	33.3% (17)	30.8% (41)	
Hypertension	50	23.5% (12)	28.6% (38)	0.49^b^
Diabetes	22	13.7% (7)	11.3% (15)	0.65^b^

^a^Mann-Whitney *U* test; ^b^Chi-square test; ^c^Student’s *t* test; *P* values ≤ 0.05 are in bold.

### The influence of preoperative radiotherapy

As shown in Table 2, the stenosis or stiffness around anastomosis was more common in patients from group PRT+ (21.1% and 0% respectively, *P* < 0.01). Correspondingly, all the loop ileostomies were closed in the patients without preoperative radiotherapy who had significantly higher reversal rates than patients from group PRT+ (100% versus 87.2%, *P* = 0.004). In group PRT+, 17 patients had eventually permanent stomas, of whom 13 patients were due to the stenosis or stiffness proximal to anastomosis (about 2 cm to 12 cm above anastomosis), 2 were due to the stenosis exactly on anastomosis, and 2 ended up with colostomy after closure of ileostomy. Median time to closure was 175 days in group PRT+, significantly longer than 119 days in group PRT− (*P* = 0.008). Difference of complications after LAR or stoma closure was not seen between two groups.

**Table 2:** The influence of preoperative radiotherapy

	PRT−	PRT+	*P* value
Reversal rates	100% (51/51)	87.2% (116/133)	**0.004**^**a**^
Time to closure, d (patients)	119 (51)	175 (116)	**0.008**^**b**^
Complication after LAR			
ileus	3.9% (2/51)	3.0% (4/133)	0.67^a^
anastomotic leakage	3.9% (2/51)	1.5% (2/133)	0.31^a^
Complication after closure			
ileus	3.9% (2/51)	6.9% (8/116)	0.73^a^
others	3.9% (2/51)	6.0% (7/116)	0.72^a^
length of stay after closure, d (patients)	8 (55)	7 (116)	0.06^b^
Stenosis or stiffness around anastomosis	0% (0/55)	21.1% (28/133)	**< 0.01**^**a**^

^a^Fisher’s exact test; ^b^Mann-Whitney *U* test; *P* values ≤ 0.05 are in bold.

### Risk factors for permanent stomas

**Figure 1:**
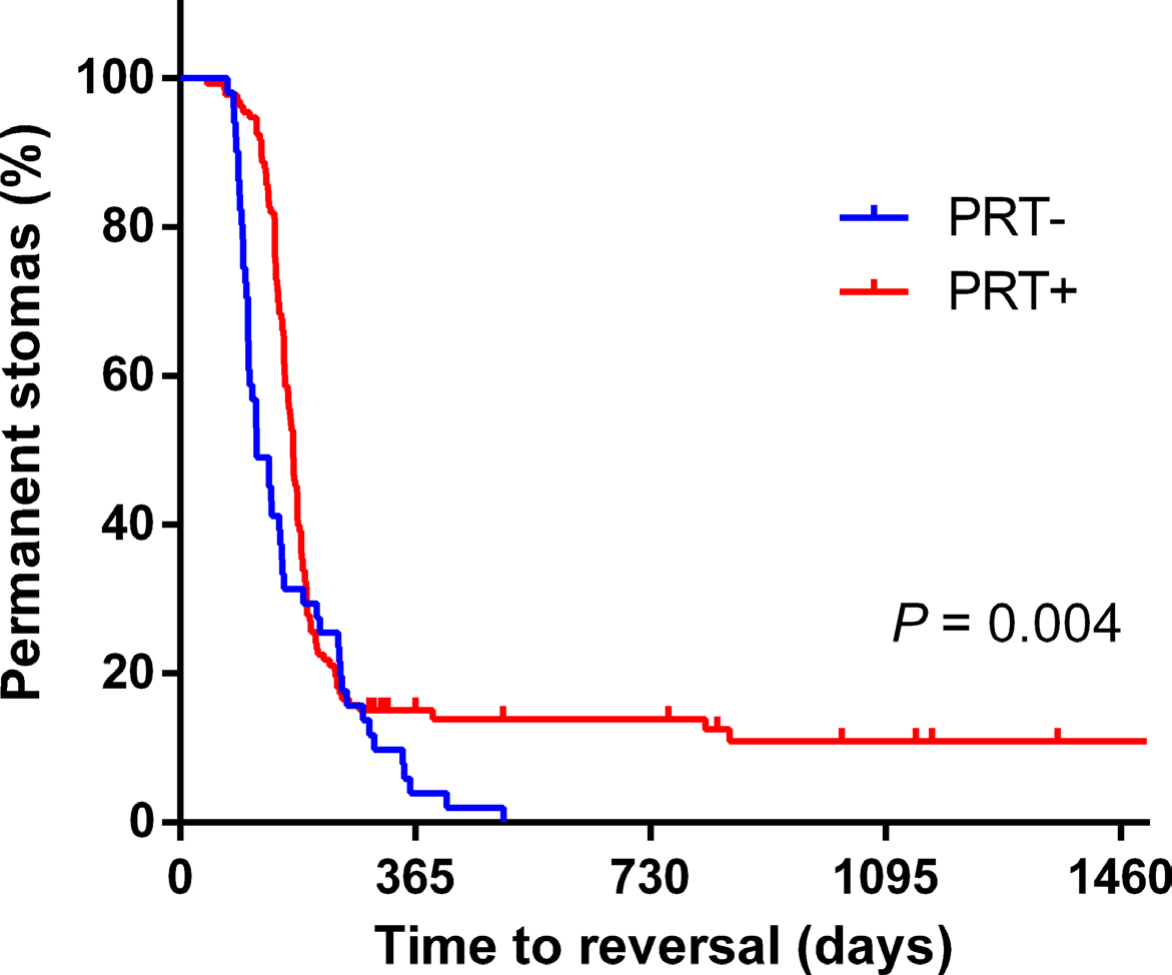
Kaplan-Meier curve for incidence of permanent stoma in PRT+ and PRT− groups

Follow-up was started from the first surgery. Median follow-up of all the patients was 567 days (range, 254–2047). 38.6% (71/184) were followed for more than two years. Two patients had a severe ileus after closure of ileostomy and received a salvage surgery of colostomy. The K-M curve showed that there were more patients received the closure of ileostomy in the PRT- group than in the PRT+ group (log-rank *P* = 0.004) (Figure 1). The following factors were brought into analysis in the multivariable Cox regression: age, sex, BMI, ASA classification, TNM tumor stage, level of anastomosis, preoperative radiotherapy, underlying diseases and rectum status around anastomosis. As shown in Table 3, tumor stage III, neoadjuvant radiation, and stenosis or stiffness around anastomosis were independent risk factors for permanent stomas.

**Table 3:** Multivariate Cox regression analysis of permanent stoma

	*P* value	HR^a^	95% CI
Tumor stage III	0.006	0.365	0.177–0.754
Preoperative radiotherapy	0.04	0.627	0.405–0.973
Stenosis or stiffness around anastomosis	0.01	0.427	0.216–0.843

^a^HR < 1 indicates higher risk of permanent stoma.

Patients with tumor stage III had a higher risk of non-reversal ileostomy (HR, 0.365; 95% CI, 0.177–0.754; *P* = 0.006). Patients with stenosis or stiffness around anastomosis were more likely suffered an extended period for closure (HR, 0.427; 95% CI, 0.216–0.843; *P* = 0.01). Patients with preoperative radiotherapy had a higher incidence of permanent stoma (HR, 0.627; 95% CI, 0.405–0.973; *P* = 0.04).

### Characteristics of anastomotic stenosis or stiffness after NCRT

**Figure 2:**
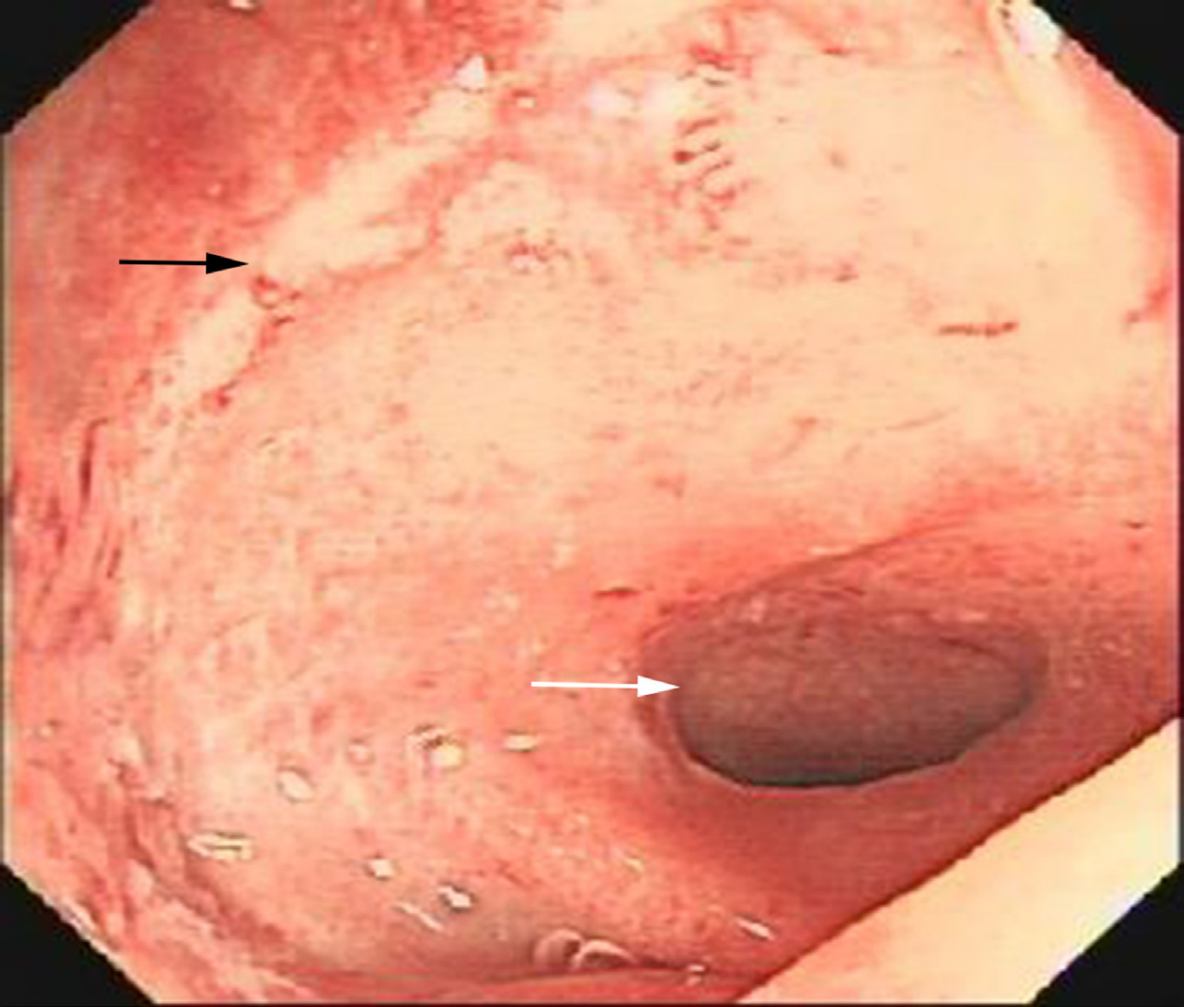
The colonoscopy showed anastomosis (black arrow) and stenosis (white arrow)

**Figure 3:**
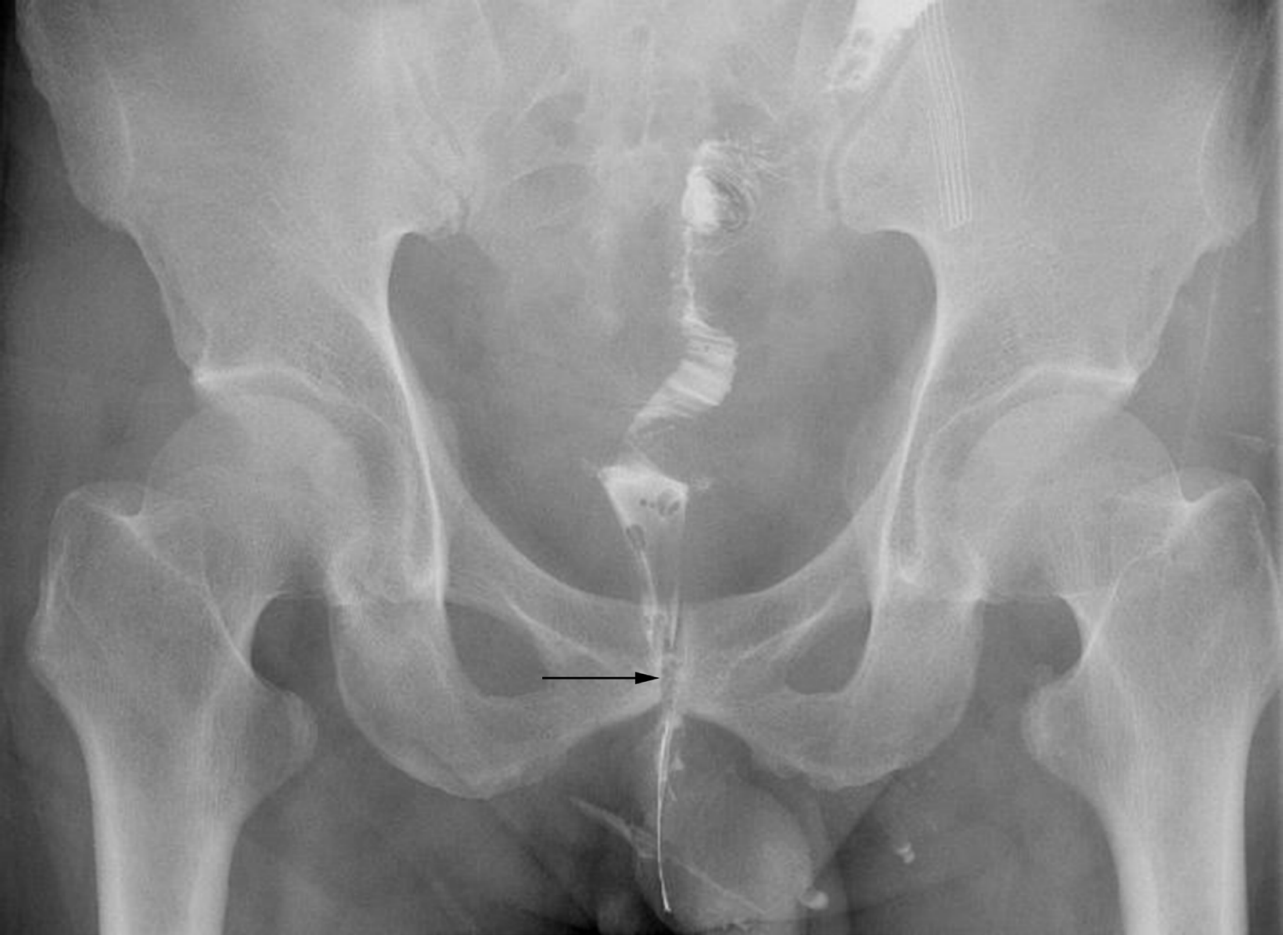
The gastrointestinal contrast showed the stenosis (black arrow) proximal to anastomosis

After NCRT and LAR, stenosis and stiffness around anastomosis would occur in some patients. In our study, most of them were positioned proximal to anastomosis. As shown in Figure 2, a colonoscopy showed a rectal stenosis 2 cm above anastomosis that prevented the passage of the colonoscope. Subsequent gastrointestinal contrast graphy confirmed the stricture proximal to anastomosis (Figure 3). Magnetic resonance imaging showed hyperplastic soft tissue in pelvic oppressing the bowel wall (figure not shown). Balloon dilatation had been performed for this kind of patients, but it was hard to relief the stenosis. And bowel wall stiffness could be described through digital rectal examination.

## DISCUSSION

Currently, low anterior resection with its sphincter-preserving function had been performed for rectal cancer more popularly. Especially for local advanced rectal cancer, in which the use of NRCT was recommended by NCCN/ESMO guidelines, LAR plus a diverting loop ileostomy was routinely used to avoid severe anastomotic complications [4, 12]. However, there was a risk of temporary stoma becoming permanent stoma for this kind of patients. Preoperative radiotherapy has been widely studied as a risk factor leading to permanent stoma, the results haven’t reached a consensus yet [8, 9, 13]. The present study demonstrated that preoperative radiotherapy had adverse effects on anastomosis and increased the rate of permanent stoma. All the patients ending up with permanent stoma were from group PRT+ (*P* = 0.004). In our study, the patients who didn’t get closure of their stoma due to the non-anastomotic reasons were excluded, thus only preoperative radiotherapy and anastomotic status would be discussed here.

Anastomotic leakage, one of the anastomotic complications, is a main factor affecting stoma reversal [9, 11]. Preoperative radiotherapy may have adverse effects on anastomotic integrity but the evidence remains conflicting and the effects may be different based on various modalities of the radiotherapy [10, 14, 15]. The radiation dose was 40–50 Gy in our study cohort. The incidence rate of anastomotic leakage was extremely low and had no statistical difference between two groups (PRT+ versus PRT**−**), which was different from another report that was also involved long-course radiotherapy [10]. Therefore anastomotic leakage was not considered to be a factor for permanent stoma in our study, thus not taken into further analysis.

Anastomotic status including stenosis should be evaluated before stoma closure. The diagnosis of stenosis was subjectively defined without unified standard in previously studies [10, 16]. In the present study, stenosis was defined as a narrow bowel lumen that prevented the passage of an 11-mm colonoscope. Several factors including anastomotic methods, surgical skills, levels of anastomotic and radiotherapy might have influence on the anastomotic stenosis [16–18]. In our study, all the patients had the same surgical procedures and stapled anastomosis. However, no anastomotic stenosis was found in group PRT- which meant weak effects of surgical factors. When controlling the confounding factors above, the results showed that preoperative radiotherapy was an independent factor for anastomotic stenosis. Remarkably, besides anastomotic stenosis, most of stenoses were positioned proximal to anastomosis, which was an evidence that the stenosis was caused by lesions in the whole pelvic region induced by radiotherapy. It had been explored that the mechanism of radiotherapy inducing stenosis might follow the process of blood vessels injury, ischemia and fibrosis [19, 20]. Such a stenosis was different from the anastomotic stricture after single surgery which was usually due to anastomotic leakage and could be easily treated with balloon dilatation [21]. Terminal ileal stricture was also a complication of radiotherapy which was poorly recognized [22]. It might partially explain the intestinal obstruction after stoma closure in group PRT+.

Besides anastomotic stenosis, anorectal radiation toxicity also included bowel wall stiffness (rectal and sigmoid wall specifically). Such stiffness was regarded as an impaired rectal distensibility induced by fibrosis [19, 23]. It might not as severe as stenosis, but still could cause fecal incontinence, tenesmus and frequency of defecation. The weakened intestinal peristalsis caused by wall stiffness might result in intestinal obstruction. All the symptoms aforesaid were possible indicators for non-reversal of stoma. Previous studies had provided limited data on the research of bowel wall stiffness and the testing methods were not optimal [24, 25]. Krol et al. used barostat to measure rectal distensibility and found the decrease of rectal cavity and compliance [23]. In the present study, although accurate function tests had not been done by technique, digital rectal examination and colonoscope did confirm the existing of bowel wall stiffness. In some patients, the bending intestine with stiff wall could even prevent the passage of colonoscope, which was a contraindication for stoma reversal. During the period of follow-up, 2 patients underwent stoma reversal at first ended up with colostomy. No stenosis but bowel wall stiffness was found in these two patients which reminded us that stoma closure might be chosen cautiously in such patients. It was interesting to point out that the bowel wall stiffness in most patients were located proximal to anastomosis. Some investigators recommended the resection of the whole intestinal segment contained in the radiation field to avoid intestinal obstruction caused by stenosis and stiffness. However, the resection range was difficult to identify and the resection only removed the risk from the bowel itself. The problem of pelvic fibrosis still could not be figured out. The mechanism of the effects of radiotherapy on colorectum and pelvic cavity needed further exploration.

No protocol of interval to closure had been set in existing reports. The loop ileostomy was planned to be reversed within 3 months but the adjuvant therapy which usually prolonged the median interval to 6 months [26–28]. In the present study, preoperative radiotherapy was a risk factor for delayed reversal which was confirmed by a previous literature [7]. One explanation was that the preoperative staging in group PRT- was obviously earlier than that in group PRT+. Some patients didn’t receive adjuvant therapy which could cut down the interval to closure.

Certainly, there were several limitations in the present study. First, its retrospective nature and small sample size would lead to bias and a less convincing result. Second, stiffness around anastomosis was a kind of subjective factor and lacked of a precise definition. Detailed analysis such as MRI scan was worthwhile. Third, the bowel dysfunction such as anorectal incontinence and diarrhea as important factors which were hard to follow up were not analyzed here.

In this retrospective cohort study, when excluding some confounding factors, 12.8% patients with preoperative radiotherapy ended with permanent stomas. The results emphasized the adverse effects of radiotherapy on stoma reversal and stenosis and stiffness proximal to anastomosis. It also provided data to support for the potential risk of permanent stoma that patients receiving preoperative radiotherapy should be informed.

## MATERIALS AND METHODS

### Study cohort

This study was approved by the ethical committee of Sir Run Run Shaw Hospital and the data were analyzed anonymously. We searched patients with rectal cancer who underwent low anterior resection (LAR) and synchronous loop ileostomy from October 2010 to December 2015 in Sir Run Run Shaw Hospital. Inclusion criteria: rectal cancer was diagnosed pathologically; total mesorectal excision (TME) was used when performing LAR; no evidence of local recurrence or distant metastasis was found during the whole study period; if the patients received preoperative radiotherapy, the radiation doses should be 40–50 Gy in 20–25 fractions. Exclusion criteria: loop ileostomy was not reversed because of factors not related to anastomosis, such as high surgical risk, economic factors and tumor progression; radiation doses were not adequate; radiation therapy was performed after LAR. Finally, 184 eligible patients were identified.

### Data collection

The data of all patients were collected at least including age, sex, body mass index (BMI), American Society of Anesthesiologists (ASA) classification, American Joint Committee on Cancer (AJCC) TNM tumor stage, location of tumor, level of anastomosis, neoadjuvant therapy and the date of surgery.

### Procedures and follow-up

133 patients with AJCC stage II and III received preoperative radiotherapy with or without concurrent chemotherapy. According to patient’s situation mFOLFOX6 was applied 1–2 cycles after the radiation therapy. A LAR with TME was performed 6–8 weeks after radiation. The other 51 patients received surgery only. All the patients were received the end-to-end anastomosis with a mechanical stapler device. A defunctioning loop ileostomy was performed simultaneously.

All patients were seen in follow-up following oncological resection. The examination including chest X-ray, liver ultrasound, tumor markers and digital rectal examination every 3 months during the first 2 years, then every six months for the next 3 years; complete colonoscopy before stoma closure or every year during the first 2 years; chest, abdominal and pelvic CT scan annually.

Anastomotic leakage was defined according to discontinuous anastomosis found by radiological or endoscopy, drainage liquid containing intestinal content and clinical symptoms of abdominal sepsis. Ileus was defined according to radiological findings and clinical signs. A stenosis around anastomosis was defined when a colonoscope with an 11-mm diameter couldn’t pass through the anastomosis or sigmoid and rectum proximal to anastomosis even after noninvasive dilatation. The stiffness around anastomosis was described as rigid bowel wall and lack of flexibility based on the report of colonoscopy or digital rectal examination. The following two conditions were defined as a non-reversal or permanent stoma: a loop ileostomy was still not reversed at the end of study; a colostomy was performed after closure of diverting ileostomy during the follow-up period.

### Statistical analysis

Chi-square test or Fisher’s exact test was used to assess difference between two groups. Student’s *t* test or Mann-Whitney U was performed to analyze continuous variables. Crude proportions of permanent stoma were presented and compared by Kaplan-Meier (K-M) method and log-rank test. Multivariable Cox regression analysis was done to confirm the independent risk factors. A two-side *P* value < 0.05 was regarded as significant. All statistical analyses were carried out by the use of SPSS statistical software (version 21.0 for Windows, SPSS, Inc.). K-M curve was plotted in Graph Pad Prism (version 6, Graph Pad, Inc.).
